# The Value of High Potencies

**Published:** 1895-02

**Authors:** Theo. H. Winans

**Affiliations:** Mexico, Mo.


### THE VALUE OF HIGH POTENCIES.

MEXICO, MO., Jan. 11th, 1895.

EDITOR HOMŒOPATHIC PHYSICIAN:

Inclosed find $2.50 for THE HOMŒOPATHIC PHYSICIAN for 1895.

I take it because you advocate high potencies as well as low. The journal or the college that doesn't do it does not teach all there is in medicine.

A young man who was on his way back to college last week, after spending the holidays at home, called on me and expressed himself on the potency question as follows: "We must use the low potencies in acute diseases. Do you hold your patients?" The college that teaches him that high potencies must not be given in acute diseases is not teaching all there is in medicine, and it throws open the doors to degenerating into old-school methods when it teaches that we *must* hold our patients. I stand with Hahnemann, and never depart from our principles to hold a patient, and my cash-book shows a few hundred dollars more this last year than the year before.

So many of these young graduates think that their pocket-books will suffer if they do not use old-school methods also. If people want allopathy they are not going to employ a homœopathist to administer it, especially where the allopaths outnumber the homœopaths ten to one, as they do in this town.

While the above-named student was in my office, it so happened that his uncle came in to report the effect of some medicine I had given him the week before. The young man listened to his exclamations of delight in so prompt relief of a bladder trouble so painful that he would lie every night for hours pressing the neck of the bladder to get relief, and asked the remedy I gave. One dose of Beer$^{dmm}$.

To counteract his teaching about not using high potencies in acute diseases, I told him of a case of dysentery where a trituration of Ip. and Merc-cor. had been given without relief, and where I gave Merc-cor.$^{cm}$ with prompt results.

A septic poisoning case, following child-birth, tenderness over whole abdomen so she could not bear pressure, lochia very offensive, cured with two or three doses Pyrogen$^{cm}$, from twelve to twenty-four hours apart. When asked to use Carbolic acid injections, or, at least, hot water injections, I told them that those organs were self-cleansing, and I allowed only cleanliness of external parts and bed, by removing frequently all soiled clothes and bathing parts with hot water. The temperature and pulse came down handsomely. There have been five deaths here of late under old-school treatment. Arsenicum$^{200}$ and Pyrogen$^{cm}$ have been my main remedies, and have cured every case that has come under my treatment since I began the practice of medicine. I may yet be able to say with Swan: "There is no need of any one's dying of blood-poisoning."

Syphilinum is one of the nosodes that has done me grand service in headache, diarrhœa, constipation, cancer, as well as cramping in the stomach until the patient doubles up (like Colocynth), and in various chronic diseases.

Talking with Dr. Crutcher, one day, about a case of cancer of the liver cured with Ars.$^{200}$, he said I should write it up to help prove the efficacy of the single remedy in high potency. An exploratory incision, four inches long, had been made in a hospital in Kansas City, over the liver, below the ribs, expecting to find a tumor. The staff of physicians all examined the exposed liver and pronounced it cancer, and said she could not live longer than two months, and she came to Mexico to die. Her friends employed me just to spend the time, because I was a homœopath and would not give her strong medicine. The liver was very much enlarged and hardened and extended well over to the left side, where the severest pains were. They might have been in the spleen. At any rate Ars. was indicated and given in the 200th potency, with prompt relief and final cure of the patient. Was it cancer? The next summer, out in Kansas, where she had gone to teach music, she contracted ague, and the old pains came back. She immediately took the train for Mexico, Mo., and put herself again under my treatment, with same results as in the previous year. She claims to be well now, but her complexion is ashy and skin dry yet.

The higher potencies have carried me through epidemics of measles, whooping cough, la grippe, and the so-called diphtheritic sore throat that has prevailed here, without the loss of a patient, while many have been lost in each of the above-named diseases by our old-school men.

In one patient only have I found the low potency to act better than the high, and that was a Podophyllum liver case of many years' standing. I could get no results until I put her on Pod.$^{3x}$ for weeks at a time, four times a day.

THEO. H. WINANS.

